# Metal Oxide Doping Modulates the Performances of Copper Oxide Nanoparticular Biocides

**DOI:** 10.3390/nano16100576

**Published:** 2026-05-08

**Authors:** Klaudia Pepłowska, Jaana Huotari, Kinga Czechowska, Marianne Vitipon, Véronique Collin-Faure, Elisabeth Chartier-Garcia, Alicja Hryniszyn, Witold Kurylak, Jacek Mazur, Satu Salo, Elisabeth Darrouzet, Adriana Wrona, Thierry Rabilloud

**Affiliations:** 1 Łukasiewicz Research Network—Institute of Non-Ferrous Metals, Sowińskiego 5 Street, 44-100 Gliwice, Poland; klaudia.peplowska@imn.lukasiewicz.gov.pl (K.P.); kinga.czechowska@imn.lukasiewicz.gov.pl (K.C.); alicja.hryniszyn@imn.lukasiewicz.gov.pl (A.H.); witold.kurylak@imn.lukasiewicz.gov.pl (W.K.); jacek.mazur@imn.lukasiewicz.gov.pl (J.M.); adriana.wrona@imn.lukasiewicz.gov.pl (A.W.); 2 VTT Technical Research Centre of Finland, Tekniikantie 21, 02150 Espoo, Finland; jaana.huotari@vtt.fi (J.H.); satu.salo@vtt.fi (S.S.); 3 Chemistry and Biology of Metals, Université Grenoble Alpes, CNRS UMR5249, CEA, IRIG-LCBM, F-38054 Grenoble, France; mariannevitipon@gmail.com (M.V.); veronique.collin@cea.fr (V.C.-F.); elisabeth.chartier-garcia@cea.fr (E.C.-G.); elisabeth.darrouzet@cea.fr (E.D.)

**Keywords:** copper oxide nanocomposites, biocide, bacteriocide, toxicity, cellular ecotoxicity, inflammation

## Abstract

Copper has been used as a biocide for more than one century, in various applications. However, as a biocide, copper, both metallic, as a salt or as copper oxide particles, is toxic not only to its intended targets, mainly bacteria and fungi, but also to all living cells. Because of this toxicity, it is desirable to use forms of copper that maximize the required biocidal activity while minimizing the amount of copper that will be released in the environment. Copper oxide nanoparticles are a good compromise for all these requirements. The high surface ratio allows for good reactivity and thus good biocidal activity, while the small amount of copper present in nanoparticles compared to microparticles allows for a limited environmental release. However, plain copper oxide nanoparticles still show significant cytotoxicity, thereby limiting their use. We, therefore, investigated if doping copper oxide nanoparticles with other metal oxide nanoparticles, namely zinc oxide or titanium dioxide, would alter the functional features of the resulting nanoparticles, hopefully increasing the biocidal activity vs. toxicity balance. We investigated biocidal activity by stringent tests using both *Staphylococcus aureus* and *Escherichia coli* as target bacteria. In addition, we investigated toxicity on mammalian macrophages or keratinocytes cell lines, as well as on an insect hemocyte cell line. Doping with zinc oxide decreased the biocidal activity, while increasing toxicity, which was the opposite of our expectations. Doping with titanium dioxide decreased the biocidal activity, but also markedly decreased cytotoxicity, which is an interesting avenue to follow. In addition, we also checked that beyond toxicity, the copper oxide-based nanoparticles did not induce an inflammatory reaction, making them safer to use.

## 1. Introduction

Copper is among the oldest biocides used on a large scale by mankind, since the pioneering work of Millardet at the end of the 19th century on the Bordeaux mixture, i.e., a fungicide made of copper sulfate and lime [1]. It has been demonstrated that, although made by the combination of two soluble solutions, Bordeaux mixture is indeed a double precipitate of hydrated copper oxide and calcium sulfate [2], which makes Bordeaux mixture one of the oldest mineral particulate biocide used. Although less popular than silver biocides in the academic context, as judged from the number of publications, copper and its derivatives are still investigated as biocides in a medical context, beside their agricultural applications. Metallic copper has demonstrated its efficiency against Gram positive (e.g., in [3]) and Gram negative [4] bacteria. It is also effective against viruses [5], including the recently infamous SARS-CoV-2 [6]. This broad activity range has led to the suggestion of using copper-based surfaces as general biocides in hospitals to reduce the bio-burden present in this environment [7].

Besides this use of plain copper-based surfaces as biocides, copper-based biocides have been used in a variety of contexts (reviewed in [8]). For example, copper oxide has been shown to be very efficient against various pathogenic bacteria, including antibiotics-resistant strains [9]. Practically, copper oxide can as be used as a deposit on non-porous surfaces [10], or in textiles [11], including wound dressings [12]. Interestingly, wound dressings including copper oxide particles have been shown to be efficient even against sporulating bacteria [13], which are very difficult to inactivate by other means.

These outstanding performances have stimulated research to understand how copper-based biocides could be both so efficient and with so little toxicity, at least on plants [14]. It was demonstrated that the micro-organisms themselves accumulated toxic intracellular doses of copper [15], as they do for other metals such as cobalt [16], cadmium [17], or mercury [18].

However, copper is not the perfect biocide, as its toxic effects, e.g., on humans, have been documented, starting with the wine workers [19]. The determination of toxic doses and the mechanisms of copper toxicity have been studied by in vitro toxicology on a variety of cell types including liver [20], kidney [21], lung [22], gut [23], skin [24], glial [25], endothelial [26], or macrophages [27] cell lines. These mechanistic studies suggest a general toxic mechanism in which the endocytosed particles are dissolved in the acidic lysosomal compartment [28], liberating free copper ion that induces oxidative stress [20,21,24] and genotoxicity [20,24,29].

Thus, research was carried out to mitigate the toxic effects of copper oxide on mammalian cells, while keeping their functional properties. As copper oxide nanoparticles are now used in many other fields than biocidal applications (see for example [30,31,32]), most of these studies have focused on the mammalian cell toxicity.

The first solution investigated was modulating the coating of the copper oxide nanoparticles [33]. However, this approach did not prove very successful, although it could be demonstrated that cationic coatings increase toxicity while coatings with reducers such as ascorbate decreased toxicity. This positive effect of such reducing coatings may also be at play in copper oxide nanoparticles made with tea extract [34], as tea extract is rich in reducing polyphenols.

Another approach has consisted of doping the copper oxide nanoparticles with other minerals. It has been demonstrated that iron doping decreases toxicity toward mammalian cells [35]. At the other end of the spectrum, copper oxide-doped titanium dioxide was evaluated as a biocide, with variable effects [36,37,38,39]. Finally, combinations of biocidal metals or metal oxides were also investigated as a way to induce a positive biocidal synergy while not inducing increased cytotoxicity toward mammalian cells. For example, a combination of copper oxide and silver showed enhanced antibacterial properties, but its toxicology was not studied [40]. As another example, a composite of copper oxide and zinc oxide was studied and shown as superior to the single element particles in its bactericidal properties, but its toxicity toward mammalian cells was not reported [41].

In fact, only a few studies report in parallel the antibacterial efficacy and toxicity (e.g., in [34,42]). This situation prompted us to carry out an evaluation of copper oxide and doped (titanium dioxide, zinc oxide) copper oxides in terms of both efficacy and toxicity.

## 2. Material and Methods

### 2.1. Nanoparticles Synthesis

#### 2.1.1. Synthesis of Copper Oxide(II) Powder

##### Materials

Copper(II) nitrate trihydrate (Cu(NO_3_)_2_·3H_2_O) (pure p.a.) and ammonium carbonate ((NH_4_)_2_CO_3_) (pure p.a.) were obtained from Chempur, Karlsruhe, Germany.

##### Experimental Method

CuO powder was produced via a two-step synthetic method. In the first step, an intermediate product was obtained by precipitation. The next step involved the thermal decomposition of the intermediate product in an argon atmosphere. Firstly, solutions of (Cu(NO_3_)_2_·3H_2_O)—(57.03 g/dm^3^) and (NH_4_)_2_CO_3_—(34.02 g/dm^3^) were prepared. Then, both solutions were added together and mixed. The precipitate formed during the process was centrifuged using a Centrifuge 5804 (Eppendorf AG) at 3500 rpm for 3 min. Then the sediment was frozen using an ULUF 65 freezer (Arctico) and dried by freeze-drying using an Alpha 2-4 LD plus (CHRIST, Martin Christ Gefriertrocknungsanlagen GmbH, Germany) freeze dryer in two stages (first stage: 96 h, −18 °C, 1.3 mbar; second stage: 2 h, −75 °C, 0.0012 mbar). The resulting material was placed in a tube furnace and heated at temperatures increasing from 100 to 300 °C over 60 min in an argon atmosphere, leading to the final CuO nanoparticles.

#### 2.1.2. Synthesis of CuO + TiO_2_ Powder

##### Materials

NaOH (granules, POCH, pure), CuSO_4_ anhydrous (Chempur, pure p.a.), TiO_2_ (Areoxide TiO_2_ P25, Evonik) were used for this synthesis.

##### Experimental Method

The CuO-TiO_2_ composite powder was chemically synthesized by precipitating CuO in the presence of suspended TiO_2_ (P25) nanoparticles. To this purpose, the following starting reagents were prepared:

(i) aqueous NaOH solutions (70 g/L, *w*/*v*) and CuSO_4_ solution (0.79 M),

(ii) aqueous TiO_2_ (P25) suspension. To this purpose, 5 g of P25 nanopowder was ultrasonically dispersed in deionized water (1.8 L) using a probe-type sonicator.

The resulting suspension was then combined and mixed with 900 mL of the NaOH solution. Subsequently, 900 mL of the copper(II) sulfate solution was added dropwise to the mixture under continuous stirring. The mixture was then heated and stirred for 1 h, ensuring that the temperature reached but did not exceed 50 °C.

After 1 h of stirring, the container with the mixture was placed in a drying chamber preheated to 50 °C and held at this temperature for 2 h. The dryer was then turned off, and after cooling to room temperature, the resulting dark precipitate was washed with deionized water to remove residual by-products, followed by centrifugation using a Centrifuge 5804 (Eppendorf SE, Germany) and freeze-drying performed with an Alpha 2-4 LDplusfreeze dryer (CHRIST).

#### 2.1.3. Synthesis of CuO-ZnO Powder

##### Materials

The CuO-ZnO powder was synthesized using copper(II) acetate monohydrate (Cu(CH_3_COO)_2_·H_2_O) (pure p.a.), zinc(II) acetate dihydrate (Zn(CH_3_COO)_2_·2H_2_O) (pure p.a.), cetyltrimethylammonium chloride (CTAB) (pure p.a.), and 25% aqueous ammonia (NH_4_OH) (pure p.a.). All reagents were obtained from Chempur.

##### Experimental Method

The CuO-ZnO powder was obtained by a single-stage chemical reaction conducted in a flow reactor with a working volume of 15 dm^3^, equipped with mechanical and magnetic stirring systems, a reflux condenser, and a system for maintaining a constant reaction temperature and pH control. The process was carried out under atmospheric conditions, without the use of a protective atmosphere. In the first stage, two precursor solutions were prepared: 250.9185 g of Cu(CH_3_COO)_2_·H_2_O and 275.887 g of Zn(CH_3_COO)_2_·2H_2_O were dissolved separately in 7 dm^3^ of distilled water each and then combined in the reactor and stirred for 5 min. Simultaneously, a dispersant solution was prepared by dissolving 0.3066 g of CTAB in 100 mL distilled water. The solution was stirred for 5 min and then sonicated for another 5 min in an ultrasonic bath (Ultrasonic cleaner Sonic-14 from Polsonic). The CTAB solution was then added to the reactor containing the precursor solutions and the stirring was continued for another 5 min. A 25% aqueous ammonia solution was then added in portions until the pH reached the value of 8. After the pH stabilized, the mixture was heated to 90 °C and held at this temperature for 2 h under constant stirring.

After the reaction was complete, the suspension was centrifuged using a Centrifuge 5804 (Eppendorf AG) at 4200 rpm for 5 min. The resulting precipitate was washed three times with deionized water (4200 rpm, 25 min) and ethyl alcohol (4200 rpm, 5 min), using 100 mL of each solvent. The purified precipitate was dried in a VACUCELL^®^ vacuum oven until a dry powder was obtained.

### 2.2. Nanoparticles Characterization

#### 2.2.1. Scanning Electron Microscopy (SEM)

The microscopic observation (SEM) was performed using a LEO Gemini 1525 high-resolution scanning electron microscope with a resolution of 1.5 nm at a voltage of 20 kV and equipped with an InLens detector. Prior to measurement, most of the samples were sputtered for 50 s with an Au conductive layer in an Ar protective atmosphere using a SEI supplies sputter coater.

#### 2.2.2. Electron Probe Microanalysis (EPMA)

Elemental analysis was performed via EPMA using an X-ray microprobe (JXA 8230 JEOL). The accelerating voltage was 15 kV. Electron photos were taken in the light of secondary and backscattered electrons. Qualitative and quantitative analyzes of chemical composition and elemental distribution maps were determined using the energy dispersion method (EDS). In the case of non-conductive powders, the samples were sputtered for 50 s with an Au conductive layer in an Ar protective atmosphere using SEI supplies sputter coater prior to measurements.

#### 2.2.3. X-Ray Diffraction (XRD)

The qualitative phase composition of powders was carried out via XRD analysis with a XRD7 diffractometer (Seifert-FPM). Characteristic X-ray radiation of Cu Kα and Ni filter were used for the investigations. Analysis was carried out in the 2θ range 10°–100°. Identification of the phases was done using Seifert and Match! software, basing the results on the ICDD PDF-4+ catalogue from 2022. Estimation of the crystallite size L was performed using Match! software using the Scherrer formula:

$$L=\frac{K\cdot \lambda }{\mathrm{FWHM}\cdot \cos\theta }$$

where K is the Scherrer constant, λ is the wavelength of the X-ray beam used, FWHM is the full width at half maximum of the peak, and θ is the Bragg angle.

#### 2.2.4. Apparent Density

The apparent density of the powders was measured by the gas pycnometry method, using a Micromeritics AccuPyc 1340 II pycnometer. Measurements were performed at room temperature in a sample chamber with a volume of 10 cm^3^ and helium as an inert gas. Results for density were obtained for 50 cycle repetitions.

#### 2.2.5. Specific Surface Area

The determination of the specific surface area S was carried out on a Micromeritics Gemini 2360 apparatus. The measuring range was 0.01 m^2^/g for the specific surface and 0.1 ÷ 300 m^2^ for the total surface. In order to remove impurities and moisture, the samples were dried overnight under conditions defined for each sample under a nitrogen atmosphere. The measurements were performed in the P/P_0_ range of 0.05 ÷ 0.30.

The average particle size $\overset{-}{d}$ was determined using the equation:

$$d=\frac{6\times 10^{3}}{\rho \cdot S}$$

where ρ is the actual density [g/cm^3^], and S is the specific surface area [m^2^/g].

### 2.3. Antimicrobial Activity

The Minimal Inhibitory Concentration (MIC) method was used [43]. To this purpose, the nano powders were dissolved to a concentration of 100 mg/mL in 3% DMSO and sonicated for 30 min at <40 °C. The Minimum Inhibitory Concentration of the dissolved nanopowders was assessed by creating a series of two-fold dilutions in sterile milliQ water. The bacterial inoculum, grown overnight in Muller Hinton Broth at 37 °C with shaking, was diluted in growth medium to a concentration of approximately 5 × 10^6^ CFU/mL. Each dilution of the nano powder was mixed with an equal volume of bacterial suspension and incubated at 37 °C for 20 h. The experiments were performed in microwell plates, with each well containing 0.2 mL and three replicates per sample. To prevent drying out during incubation, the plates were placed in a plastic container with water at the bottom. Following the incubation period, microbial survival was determined by culturing 10 µL of each dilution on Plate Count agar and incubating at 37 °C for 24 h. The test bacteria used were *Staphylococcus aureus* VTT E-70045 and *Escherichia coli* VTT E-94564 (VTT Culture Collection, VTT Technical Research Centre of Finland Ltd., Espoo, Finland).

### 2.4. Nanotoxicology

#### 2.4.1. Particles Dispersion for Nanotoxicology

As the cell culture cannot tolerate a high concentration of DMSO, an aqueous resuspension medium was selected for these experiments. The nanoparticles were thus dispersed at 10 mg/mL in an aqueous solution of hydroxypropylmethylcellulose (Sigma # 423238, 5 mg/mL), previously sterilized overnight at 80 °C in a humid atmosphere. Nanoparticles dispersions were then re-sterilized under the same conditions to minimize microbial contamination. To reduce agglomerates and aggregates, the dispersions were sonicated in a Vibra Cell VC 750 sonicator (VWR, Fontenay-sous-Bois, France) equipped with a Cup Horn probe. Sonication was carried out in pulse mode for 30 min (1 s ON/1 s OFF) at 60% amplitude, corresponding to 90 W per pulse, in volume not exceeding 3 mL to ensure efficient energy transfer. Dispersions were stored at 4 °C. Prior to use, pigment dispersions were sonicated for 15 min in an ultrasonic bath, then diluted in sterile water to intermediate concentrations as required.

For the in-solution characterization of the nanoparticles dispersions, Dynamic Light Scattering (DLS) was performed using a Litesizer 200 instrument (Anton Paar, Les Ulis, France) equipped with an Omega reusable cuvette (225288, Anton Paar). Nanoparticles dispersions were diluted to a final concentration of 10 or 50 µg/mL in 0.001× PBS to ensure adequate particle presence for detection while maintaining optimal optical transmittance. DLS measurements were conducted at 25 °C. An average value was obtained from repeated measurements for each sample (n = 3) and analyzed with the instrument-associated Kaliope software. The hydrodynamic size distribution was expressed as number-weighted intensity.

#### 2.4.2. Mammalian Cells Toxicity Assay

To evaluate the toxicity of the nanoparticles, cell survival assays were performed, using the MTT reduction method [44]. As target cell lines, we used the J774A.1 murine macrophage cell line [45] and the HaCat human keratinocyte cell line [46]. The J774A.1 cell line was purchased from ECACC (Salisbury, UK), while the HaCat cell line was a gift of Pr N. Fusenig (Heidelberg, Germany). Both cell lines were cultured in DMEM supplemented with 10% fetal bovine serum.

For routine culture, the J77A.1 cells were seeded at 200,000 cells/mL in non-adherent cell culture flasks (Greiner) and harvested two days later at 800,000–1,000,000 cells/mL by tapping the flask. The HaCat cells were seeded in adherent culture flasks (Falcon) at 20,000 cells/cm^2^ and recovered at confluence 3 days later. For passaging the HaCat cells, the cell layer was rinsed three times in serum-free DMEM, and then incubated in trypsin EDTA (0.25% trypsin, 1 mM EDTA in PBS) at a ratio of 0.05 mL/cm^2^ at 37 °C for 5–10 min. The reaction was stopped by diluting the cell suspension in the trypsin solution into 5 volumes of complete culture medium (with serum). The cell suspension was then centrifuged (200× *g*, 5 min) to pellet the cells, which were then resuspended in complete culture medium, numerated using the Trypan blue method [47] and diluted for culture.

For the survival assay tests, the cells were seeded in 24 wells adherent culture plates at a density of 500,000 cells/mL for J774A.1 cells and 80,000 cells/cm^2^ for HaCat cells. After 24 h, the nanoparticles suspensions were added to the wells and the cells returned to the cell culture incubator for 24 h. At the end of the exposure period, the culture medium was removed, the cells layers were rinsed once with DMEM without phenol red and then incubated for 1 h at 37 °C in serum-free and phenol red-free DMEM containing 0.2 mg/mL MTT (predissolved at 20 mg/mL in ethanol). At the end of the MTT exposure period, the culture medium was removed and the produced formazan eluted in a solution containing 90% ethanol and 1% acetic acid in water (all by volumes). The plates were placed on a plate shaker for 30 min at room temperature. The eluate was transferred to a fresh plate to avoid interferences due to the opacity of the added nanoparticles, and the absorbance at 570 nm was measured by a BMG Fluostar Omega plate reader.

In order to better mimic occupational exposure, a repeated exposure scheme was also used. In this scheme, the cells were seeded in culture medium containing 1% horse serum instead of 10% fetal bovine serum to limit cell proliferation [48]. After 24 h, a split dose, i.e., a quarter of the final cumulated dose was added to the medium. After 24 h of exposure the medium was changed, and a split dose was added. After 24 h another split dose was added. Finally, the medium was changed, and a fourth split dose was added, before a final 24 h culture period. The medium was then removed, and the cell viability testing protocol was carried out as described above.

#### 2.4.3. Cytokine Release Assays

In order to evaluate the pro-inflammatory secretion of cytokines induced by the nanoparticles, the cell supernatants collected after the acute exposure to the nanoparticles were centrifuged at 15,000× *g* for 30 min to remove the nanoparticles, and then assayed for the presence of Interleukin 6 (IL-6) and Tumor Necrosis Factor alpha (TNF). The chemokine MCP1 (for J774A.1 cells) and Interleukin 8 (IL-8, for HaCat cells) were also measured. The cytokine bead array method was used for the multiplexed measurements of the cytokines, using the Human Inflammatory Cytokine kit (BD biosciences, #551811 for the HaCat cells and the Cytometric Bead Array Mouse Inflammation Kit (catalog numbers 558299, 558301, and 558266, BD Biosciences) for the J774A.1 cells.

#### 2.4.4. Phagocytosis Assay

This test was performed only on J774A.1 cells, as these cells are known to be phagocytic. After acute exposure to the nanoparticles, the cells were exposed for 3 h at 37 °C to latex beads (carboxypolystyrene, yellow-green labelled, 0.5 µm, #15700 from Polysciences). The cells were then rinsed twice with PBS, and analyzed for the green fluorescence (excitation 488 nm, emission 527/32 nm) on a Melody flow cytometer. Controls with no latex beads showed no green fluorescence.

#### 2.4.5. Toxicity on Insect Cells

The toxicity test was performed on Schneider S2 cells, which are a model for insect hemocytes [49]. For culture maintenance, the cells (purchased from DSMZ, Germany) were grown at 25 °C in T75 flasks in an air atmosphere and in a culture medium composed of 4.5 parts of Schneider medium, 4.5 parts of TC100 medium, and 1 part of fetal bovine serum. Cells were routinely seeded at 40,000–80,000 cells/cm^2^ and harvested by scraping after 2 to 4 days.

For toxicity testing, the cells were seeded at 250,000 cells/cm^2^ in 24 well culture plates. After 24 h of settling and growth, the cells were treated with varying concentrations of the nanoparticles for 24 h. After this incubation period, the cell viability was assessed with a WST1 viability kit (# 11644807001 from Sigma Aldrich Millipore, Saint Quentin Fallavier, France) with a dilution factor of 1:10 and an incubation period of 4 h. At the end of the incubation period, the plates were centrifuged (200× *g* for 5 min) and the supernatant collected for analysis by absorbance at 440 nm. The cell layer was then fixed with 50% ethanol for 40 min, and the ethanol was removed. The cell layer was then rehydrated in culture medium overnight, and a second WST1 test was performed to determine the assay background, which was then subtracted from the cell assay to obtain the corrected absorbance.

## 3. Results

### 3.1. Nanoparticles Characterization

#### 3.1.1. Scanning Electron Microscopy (SEM)

**Figure 1:**
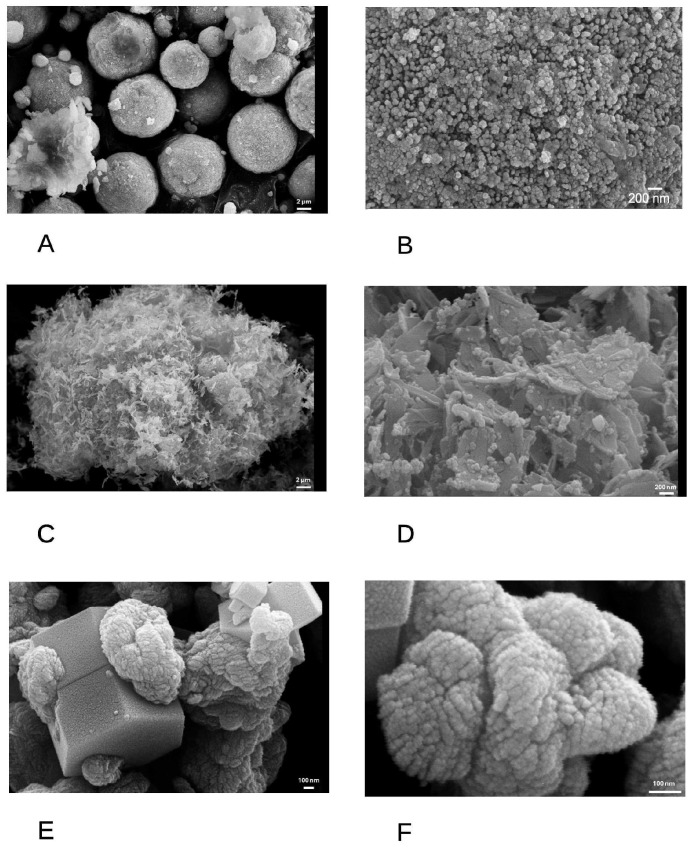
Microstructure of the nanoparticle agglomerates. (**A**,**B**) CuO powder agglomerates Magnification 5000× and 50,000×, respectively, (**C**,**D**) CuO–TiO_2_ powder agglomerates. Magnification 5000× and 50,000×, respectively, (**E**,**F**) CuO–ZnO powder agglomerates. Magnification 100,000× and 300,000×, respectively.

The three nanoparticles produced for this study were first characterized by scanning electron microscopy. The results, displayed on Figure 1, showed very different morphologies for the three particles.

The CuO nanopowder consisted of spherical aggregates (Figure 1A), made themselves of very small (ca. 20 nm) primary particles (Figure 1B). The CuO-TiO_2_ composite consisted of flaky aggregates (Figure 1C), made themselves of CuO flakes on which spherical TiO_2_ nanoparticles (ca. 20 nm in diameter) were deposited (Figure 1D). This showed that the presence of relatively minute amounts of TiO_2_ nanoparticle greatly altered the CuO precipitation process. The CuO-ZnO composite also showed a complex structure, combining ZnO plates on which irregular, dense CuO agglomerates were deposited (Figure 1E,F).

#### 3.1.2. Electron Probe Microanalysis (EPMA)

Electron probe microanalysis allowed to determine the presence of the various elements and their respective percentages in the nanomaterials produced for this study. The results, displayed in Table 1, showed that the preparation process did not induce a mineralogical bias in the final nanomaterials compositions compared to the respective metal inputs used for the synthesis for the CuO-TiO_2_ nanocomposite. For the CuO-ZnO nanocomposite, the final product was enriched in copper compared to zinc (3:1 metal ratio), although the metal input was 1:1 at the beginning of the synthesis. This showed a higher precipitation efficiency for CuO than for ZnO in the synthetic conditions used.

#### 3.1.3. X-Ray Diffraction (XRD)

**Figure 2:**
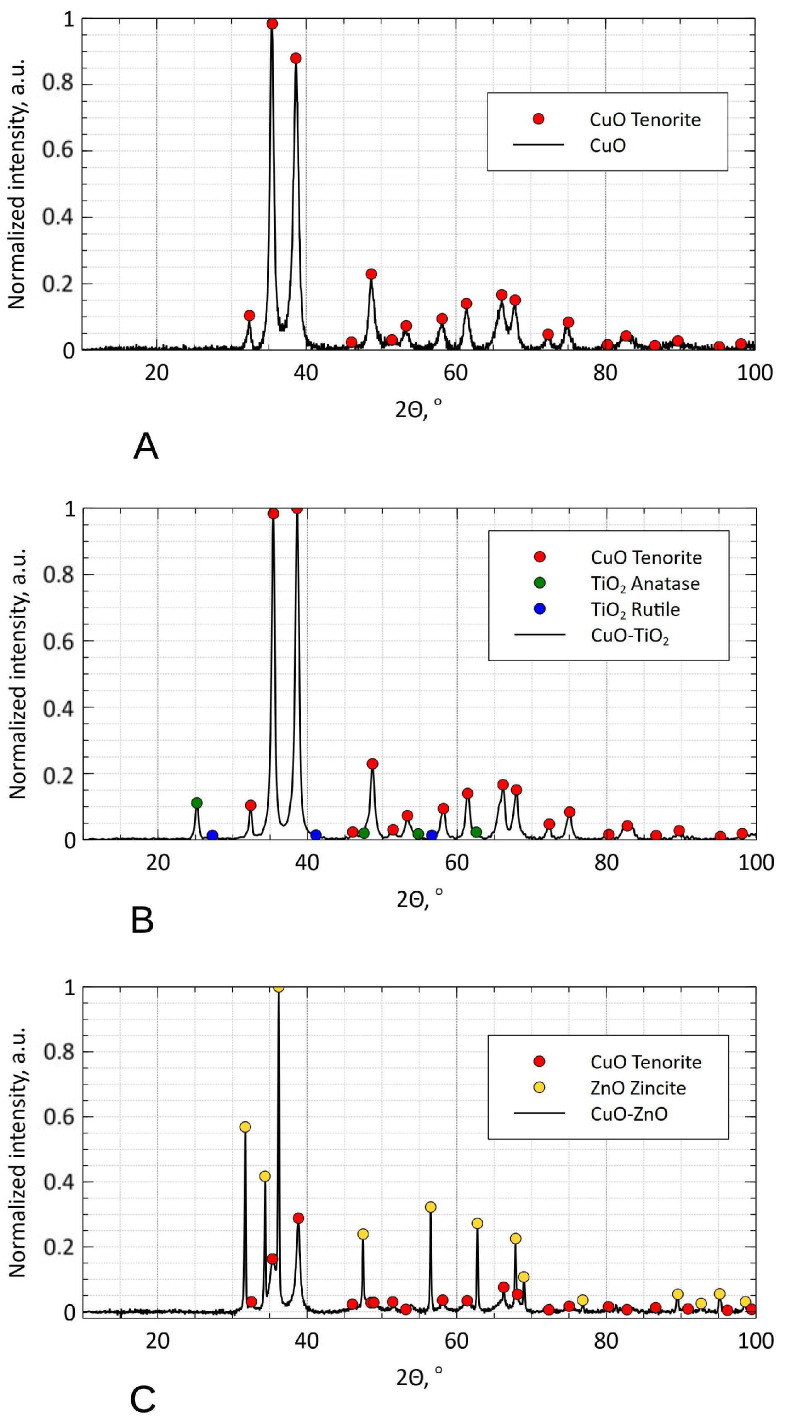
X-ray diffraction patterns of the nanoparticles. (**A**) X-ray diffraction pattern of the copper(II) oxide powder. (**B**) X-ray diffraction pattern of the copper(II) oxide-titanium(IV) oxide powder. (**C**) X-ray diffraction pattern of the copper(II) oxide-zinc(II) oxide powder.

X-ray diffraction was used to characterize the mineralogical phases present in the final nanomaterials. The XRD diagrams are displayed in Figure 2. In all cases, copper oxide was present as tenorite (e.g., in Figure 2A). In addition to tenorite, rutile and anatase were also detected in the CuO-TiO_2_ nanomaterial (Figure 2B), which is consistent with the crystalline phases present in the Aeroxide starting material. Finally, the zinc oxide phase present in the CuO-ZnO composite was assigned to zincite (Figure 2C).

#### 3.1.4. Apparent Density, Specific Surface Area, Average Particle Size

As additional characterization parameters, we measured the apparent density and the specific surface area, which allowed to derive the average particle sizes. The results are displayed in Table 2.

The specific surface area of the CuO nanomaterial was almost twice higher than for the nanocomposites, which is consistent with the surface structure observed by SEM. Indeed, the primary particle size derived from BET is quite similar to the one observed by SEM (<20 nm). Interestingly, the specific surface of the nanocomposites was quite similar, although their structure as revealed by SEM was very different.

#### 3.1.5. Nanoparticles Dispersion in Aqueous Media

The hydrodynamic diameter data obtained on the nanoparticle dispersions in the HPMC solution are summarized in Table 3.

Compared to the results obtained on the nanopowders, these results show that the dispersions are composed mostly of particles agglomerates, which is quite common for copper oxide nanoparticles [27].

### 3.2. Antibacterial Efficacy

The antibacterial efficacy was determined by the MIC method, and the results are summarized on Table 4. Three independent experiments were performed, and their individual results are presented

These data show that particle doping greatly increased the MIC compared to plain copper oxide nanoparticles, which means a reduced antimicrobial efficacy.

### 3.3. Biological Effects on Mammalian Cells

The biological effects were investigated first on a macrophage cell line (J774A.1), as macrophages are scavenger cells positioned on multiple localizations in the body and handle all types of particulates that may enter into the body or be inhaled in the lungs. We also investigated the biological effects on a keratinocyte cell line (HaCat) in order to obtain an appraisal of the potential effects on skin.

**Figure 3:**
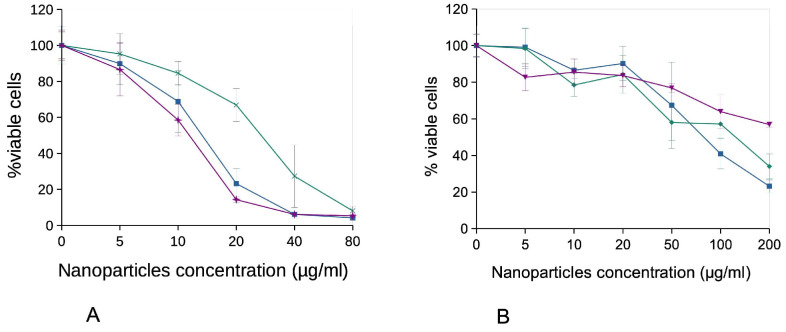
Survival curves of cells treated for 24 h with the copper oxide-based nanoparticles. J774A.1 cells (**A**) and HaCaT cells (**B**) were treated at confluence for 24 h with copper oxide-based nanoparticles in complete cell culture medium (containing 10% FBS). The cell viability was then measured by the MTT assay. The results are displayed as mean ± standard deviation (N = 4). Blue curve: cells exposed to CuO. Green curve: cells exposed to CuO-TiO_2_. Purple curve: cells exposed to CuO-ZnO.

We first investigated the effects on cell viability. The results, displayed on Figure 3, showed a greater toxicity for macrophages than for keratinocytes, which can be explained by the phagocytosis of the nanoparticles agglomerates by the macrophages, keratinocytes being non-phagocytic. In the case of macrophages, the CuO-TiO_2_ composite nanoparticles proved much less toxic than the other two, with a LD50 close to 30 µg/mL instead of 13–14 µg/mL for the other two particles. For keratinocytes, the toxicity of the three particles was very similar, with LD50 in the 100–200 µg/mL range.

**Figure 4:**
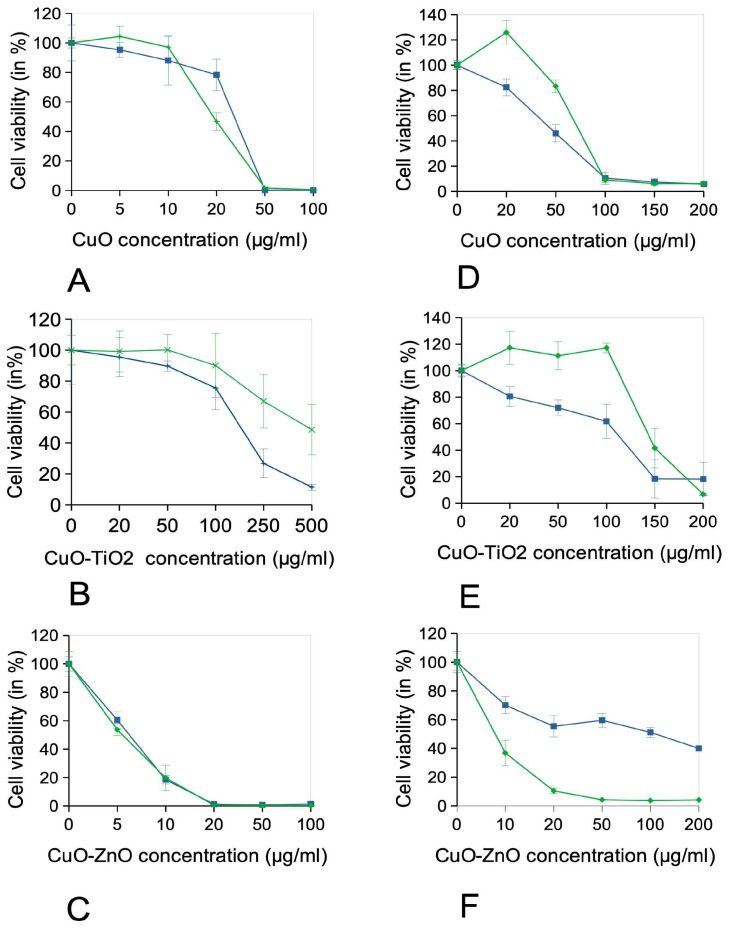
Survival curves of cells treated for 4 days with the copper oxide-based nanoparticles. J774A.1 cells (**A**–**C**) and HaCaT cells (**D**–**F**) were treated at confluence either for 24 h or 4 days with copper oxide-based nanoparticles in adapted cell culture medium (containing 1% horse serum). The indicated dose is the cumulated exposure concentration. The cell viability was then measured by the MTT assay. The results are displayed as mean ± standard deviation (N = 4). Blue curve: acute exposure. Green curve: repeated exposure.

In order to obtain a better appraisal of the toxicity of the nanoparticles, we then investigated their toxic effects in a repeated exposure mode, where the total dose is fractionated in four daily doses. This allows to better evaluate the effects of the biocides on workers, which may be exposed repeatedly to sub-toxic doses. The results, displayed on Figure 4, showed almost no difference for the two exposure modes for CuO on both cell lines, and for CuO-ZnO for macrophages. CuO-ZnO proved more toxic in the repeated exposure mode for keratinocytes, while less toxicity was observed for CuO-TiO_2_ in the repeated exposure scheme on both cell lines.

A lesser toxicity in the repeated mode can be attributed to the fact that cells exposed to sub-toxic doses are able to build a protective response against further exposure, while a higher toxicity in the repeated mode can be attributed to the fact that the nanoparticles show a delayed toxicity that develops over time, even at a dose that is not toxic after 24 h. It should also be noted that the survival curves shown for acute exposure may differ from those shown in Figure 3, as the culture conditions are different. Some conditions also show a higher MTT signal than the control, which translates in apparent viabilities higher than 100%. This is due to the fact that the MTT assay is a metabolic assay, so that cells exposed to non-toxic particles concentrations may be subject to metabolic activation in order to fight this stress.

Beyond cell toxicity, particles can induce adverse effects via inflammation, as documented for silicosis and asbestosis. We thus investigated the production of pro-inflammatory cytokines by cells treated with non-toxic concentrations of the particles. The results, displayed on Table 5 and Table 6, showed no induction of the secretion of interleukin 6 and sometimes even a significant decrease, opposite to what has been described on the J774A.1 line for amorphous silica, for example [50].

For J774A.1, a decrease, sometimes significant, was also observed for the MCP-1 chemokine. The situation was different for TNF, where a small (ca. 1.2-fold) but significant increase was observed in response to CuO and CuO-TiO_2_ particles, while a decrease was observed in response to CuO-ZnO. However, this increase is much lower compared to the one observed in response to amorphous silica [50].

Regarding the HaCaT cells, we measured the human chemokine interleukin 8 in place of MCP-1, and also IL-6. We could not detect any TNF secretion in any of the tested conditions.

No significant change was observed for IL-6 secretion. However, a significant increase in the secretion of IL-8 was observed in response to all three nanoparticles.

**Figure 5:**
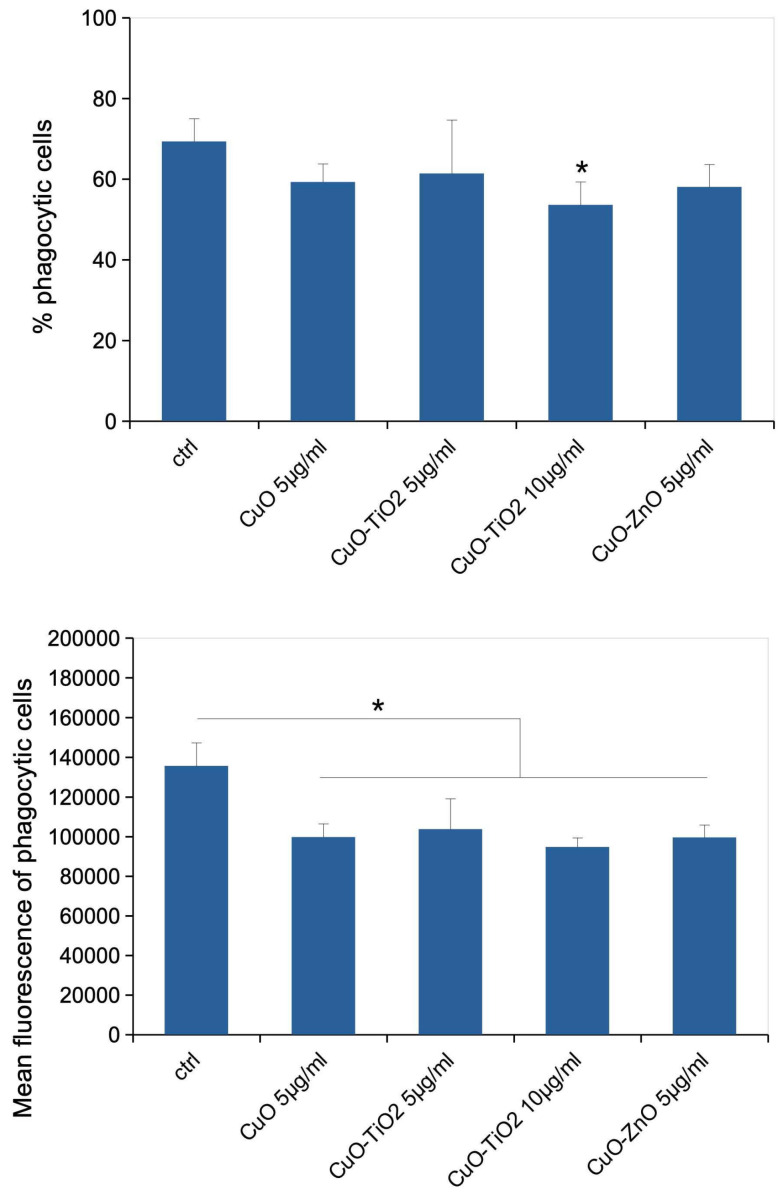
Phagocytic activity of J774A.1 cells treated for 24 h with copper oxide-based nanoparticles. In the (**top**) panel, the proportion of phagocytosis positive cells (i.e., cells having internalized at least one latex bead in three hours) is shown. The (**bottom**) panel shows the amount of fluorescence due to beads internalization within the three hours phagocytosis window. Results are shown as mean ± standard deviation. Significance mark: * = *p* < 0.05 (Student *T* test, N = 4).

Last but not least, we investigated whether the copper oxide-based nanoparticles, when present at a non -toxic dose, could alter the phagocytic capacity of J774A.1 cells, as phagocytosis is an important function of macrophages. The results, displayed on Figure 5, showed a slight (one third) but significant decrease in the phagocytic capacity of J774A.1 cells in response to the three nanoparticles tested.

### 3.4. Biological Effects on Insect Cells

**Figure 6:**
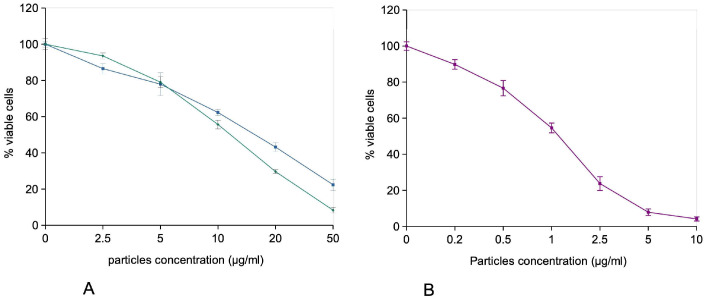
Survival curves of cells treated for 24 h with copper oxide-based nanoparticles. Schneider cells were treated at near confluence for 24 h with copper oxide-based nanoparticles in complete cell culture medium (containing 10% FBS). The cell viability was then measured by the WST1 assay. The results are displayed as mean ± standard deviation (N = 4). (**A**) cells exposed to CuO and CuO-TiO_2_ particles. Blue curve: cells exposed to CuO. Green curve: cells exposed to CuO-TiO_2_. (**B**) Purple curve: cells exposed to CuO-ZnO.

In order to get a first appraisal of ecotoxicity, we tested the effect of the copper oxide-based biocides on Schneider cells, which are a model of insect hemocytes [48]. The results, displayed on Figure 6, showed a higher toxicity for the Cuo-ZnO nanoparticles (LD50 around 1.2 µg/mL) than for CuO and Cuo-TiO_2_ nanoparticles (LD50 around 16 and 12 µg/mL, respectively).

## 4. Discussion

When designing/optimizing a biocide, several parameters must be taken into account. The first parameter is of course efficacy. It is indeed a multifactorial parameter. It includes of course the efficient doses, but also the spectrum of inhibition (i.e., the range of micro-organisms against which the biocide works) and the duration of the inhibition over time. Mineral biocides in general and copper-based biocides in particular have demonstrated their efficacy, as they inhibit the growth of various bacteria [9], including spore forming bacteria [13], as well as fungi and viruses [8]. Moreover, mineral biocides are fairly resistant to weathering, which ensures a persistence of the biocidal effect over time, as the Bordeaux mixture example shows.

However, biocides are not designed drugs, and, therefore, often act by very generic pathways such as oxidative stress [20,21,22,24] or wide-scope enzymes inhibition [51,52]. This explains why efficacy is often counterbalanced by toxicity to untargeted organisms, such as mammals or other eukaryotes. Thus, the counterpart of the efficacy of the mineral biocides is their widely documented toxicity toward humans (see for example [19]) or other organisms [53]. Thus, there is a balance to find between these two contradictory requirements.

Last but not least, biocides are industrial products, so that their production should be compatible with an industrial scale.

With all these requirements in mind, we tried to compare our results with those previously published in the literature. Regarding the bactericidal efficacy, the comparison was made difficult by the variety of techniques used to measure this parameter. Some authors, like us, use the serial dilution method to determine the minimal inhibitory concentration (MIC) (e.g., [9,34]). Others use another serial dilution method to determine the minimal bacteriostatic concentration (MBC) (e.g., in [40]). Others use the well diffusion method, in which a clear halo devoid of bacteria appears around wells loaded with the biocide, against the bacterial layer grown on a bacterial growth plate (e.g., [54,55,56]). Even worse, changes in the experimental conditions within the same method can drastically alter the outcome of the test. As Ren et al. [9], we used in our MIC tests bacterial inocula in millions of live bacteria per ml. Other scientists working in microplate format used only thousands of live bacteria per ml [34]. This difference in inoculum concentration makes of course total bacteria killing much easier when less bacteria are present at start, leading to lower MICs.

Thus, the only comparable reference values that we could find in the literature are those from Ren et al. [9]. As expected, the MIC values that we found for plain copper oxide nanoparticles and theirs were in the same range. Regarding the CuO-ZnO composite, the only paper reporting its use did not test the free particles dispersion but a particle impregnated fabric [41]. As this ensures maximal nanoparticles dispersion, opposite to what can be achieved by powders sonication in solvents, it is not surprising that they report extremely low MICs in their system (µg/mL instead of mg/mL in our case).

For the CuO-ZnO composite, we had to use a dispersant (CTAB) to avoid macroprecipitation of the material. However, CTAB is an antibacterial by itself [57], and is also toxic for mammalian cells [58]. We had thus to figure out whether remaining CTAB could play a role in the observed biological effects. Regarding the bactericidal effect, if remaining CTAB had any effect, we should observe a decreased MIC in our tests, which is clearly not the case. For the toxicity toward animal cells, it has been described that CTAB is not cytotoxic up to millimolar concentrations [59,60]. When calculating the simple dilution of the excess detergent by our washing process, the remaining CTAB concentration should be below the 1mM threshold, i.e., below the toxic level. Indeed, toxicity of CTAB in nanomaterials has been described (e.g., in [61]) but for syntheses that used a much higher concentration of CTAB [62]. As the toxicity of CTAB in nanomaterials preparation has been associated with free CTAB remaining in the synthetic mix [63], the extensive washings performed in our synthesis rule out such a possibility. This hypothesis is also substantiated by the fact that the LD50 observed for the CuO-ZnO composite (13 µg/mL) is quite close to the one observed for CTAB-free ZnO nanoparticles [64]. Moreover, should the effect of the CuO-ZnO composite be partly explained by remaining CTAB, a lower MIC should have been observed too, which is not the case.

Finally, no reference values could be found for our CuO-TiO_2_ composite, as our composition was very far from those described in the literature, in which TiO_2_ makes the bulk of the material [36,37,38,39]. Thus, the values that we report may be conservative, but are obtained under the most demanding conditions.

Regarding the toxicity toward mammalian cells, plain CuO was also used as a reference compared to the literature. As the toxic threshold can drastically vary from one cell type to another, we had to compare our values to those published on similar ones. Regarding macrophages, our values were quite similar to those published on other macrophage cell lines [27,33]. The situation was however quite different for keratinocytes, where we did not detect a strong cytotoxicity up to 100 µg/mL, opposite to previous publications [24], which detected a strong cytotoxicity above 30 µg/mL. Once again, experimental details may explain this discrepancy. Alarifi et al. described nanoparticles in the 40–60 nm range, while our copper agglomerates in water-based media are in the 200 nm range. While this does not make any difference for phagocytic cells such as macrophages, which engulf any particle up to several microns [65], this may make a difference for non-phagocytic cells, which do not easily internalize particles bigger than 100 nm (e.g., in [66]). Thus, the final internalized dose in keratinocytes may drastically differ, depending on the actual size of the nanoparticles dispersions present in the culture medium, resulting in different observed toxicities.

In order to get a first appraisal of ecotoxicity, we selected insect cells because insects are widespread and at the basis of many food chains. Hemocytes were then selected because they are the functional homologs of human macrophages. Moreover, in an ingestion model, nanoplastics have been shown to translocate through the intestinal barrier, reach and impact hemocytes [67,68]. Hemocytes are the main cell component of insects’ immunity [69], but their functions seem to extend well beyond immunity [70], making them an attracting target to study the impact of biocides. It should be noted that in our study, the insect cells appeared more sensitive to the toxic effects of the biocides than mammalian cells, with LD50 around 12–15 µg/mL for Cuo-TiO_2_ and CuO, respectively, instead of the 15–30 µg/mL observed for macrophages. The results were even more different for the CuO-ZnO composite, for which the LD 50 was only 1.2 µg/mL for the insect cells, instead of 13 µg/mL for murine macrophages. This may be due to the smaller size of insect cells, but also to more specific effects that are not yet known.

Last but not least, we took great care to select synthetic processes that could be upscaled easily. There has been a blossoming of “green” synthetic processes using plant extracts to synthetize copper oxide nanoparticles (e.g., in [34,55]) or of processes using ultrasound to produce these particles (e.g., in [42]). For the time being, these processes have not demonstrated their scalability. Furthermore, the copper oxide nanoparticles produced by these processes were not less toxic toward epithelial cells than ours.

In this frame, when examining the interest of doping of copper oxide nanoparticles with other metal oxides, we observed that composites showed a lesser biocidal efficacy compared to plain copper oxide nanoparticles. This correlated well with the surface area of the nanomaterials, as it is well known that an increase in the surface area correlates with a higher bactericidal activity [71]. As the same parameter is at play for the toxicity of copper oxide toward animal cells [72], it is not surprising that copper oxide is more toxic, than the CuO-TiO_2_ nanocomposite, although the latter is made of CuO for 92% and of only 8% TiO_2_. The situation is different for the CuO-ZnO composite, which is made of ca. 75% of copper oxide and 25% of zinc oxide. We observed that doping by zinc oxide induced strong cytotoxicity, especially for epithelial cells under the repeated exposure scheme and for insect cells. This may be due to the conjunction of the very easy dissolution of zinc oxide in biological media, especially in the acidic environment of the cellular lysosomes [73], coupled with its strong cytotoxicity for keratinocytes [74]. Even for macrophages, we observed that zinc oxide [64] was more toxic than copper oxide [27]. Conversely, doping with titanium dioxide decreased cytotoxicity, especially in the repeated exposure scheme. However, it also decreases the antibacterial performances, both correlating with the lower surface area of the particles compared to CuO. Nevertheless, we believe that titanium dioxide doping may be useful to design safer and still efficient biocides in the future.

In conclusion, we believe that the main value of this work is to show the interest of an integrated work, which couples the production of new nanocomposites (and the possibilities are almost infinite) to the evaluation of their performances, both positive (e.g., bactericidal activity) and negative (toxicity and ecotoxicity for different cell types). As described above, this integrated approach provides a corpus of information that is required to make decisions when carrying on the development of new mineral biocides.

## Figures and Tables

**Table 1:** Elemental composition of the CuO, CuO-TiO_2_ and CuO-ZnO.

Element	CuO	CuO-TiO_2_	CuO-ZnO
	wt%		
O	16.9 ± 1.3	17.7 ± 1.7	12.8 ± 2.1
Ti	-	5.2 ± 1.3	-
Zn	-	-	21.8 ± 2.1
Cu	83.1 ± 1.3	77.1 ± 2.5	65.5 ± 9.2
	wt% recalculated as oxides		
CuO	100	91.8	75.2
TiO_2_		9.2	
ZnO			24.8

**Table 2:** Physical properties of the nanomaterials.

Average CuO Crystallites Size dXRD, nm	Average Particle Size dBET, nm	Specific Surface Area m^2^/g	Apparent Density g/cm^3^	Material
13	17	62.51	5.85	CuO
19	29	36.18	5.79	CuO-TiO_2_
13	33	34.76	4.99	CuO-ZnO

**Table 3:** Parameters of the aqueous dispersion of the copper oxide-based nanoparticles.

Particle	Hydrodynamic Diameter	Polydispersity Index
CuO	216 ± 14 nm	27.6 ± 1
CuO-TiO_2_	553 ± 15 nm	21.7 ± 1.4
CuO-ZnO	374 ± 2 nm	9.6 ± 2.5

**Table 4:** MIC for the copper oxide-based nanoparticles.

	Experiment 1	Experiment 2	Experiment 3
	MIC (mg/mL)	MIC (mg/mL)	MIC (mg/mL)
		*S. aureus*	
CuO	6.25	6.25	6.25
CuO-TiO_2_	50	>50	>50
CuO-ZnO	12.5	25	25
		*E. coli*	
CuO	0.78	0.78	1.56
CuO-TiO_2_	25	25	25
CuO-ZnO	25	25	50

**Table 5:** Cytokine secretion in J774A.1 cells exposed to nanoparticles.

	IL-6	MCP-1	TNF α
CuO 5 µg/mL	0.53 ± 0.07 ***	0.83 ± 0.01 **	1.12 ± 0.14
CuO 10 µg/mL	0.45 ± 0.09 ***	0.88 ± 0.06 *	1.25 ± 0.14 *
CuO-TiO_2_ 5 µg/mL	0.79 ± 0.11 *	0.91 ± 0.09	1.21 ± 0.09 *
CuO-ZnO 5 µg/mL	0.43 ± 0.08 ***	0.77 ± 0.02 ***	0.77 ± 0.13 *

The results are expressed as fold change compared to the control condition (mean ± standard deviation). Significance marks: * = *p* < 0.05; ** = *p* < 0.01; *** = *p* < 0.001 (Student *T* test, N = 4).

**Table 6:** Cytokine secretion in HaCaT cells exposed to nanoparticles.

	IL-6	IL-8
CuO 20 µg/mL	1.02 ± 0.11	1.49 ± 0.08 ***
CuO-TiO_2_ 10 µg/mL	1.09 ± 0.09	1.25 ± 0.12 *
CuO-ZnO 10 µg/mL	1.03 ± 0.08	2.45 ± 0.21 ***

The results are expressed as fold change compared to the control condition (mean ± standard deviation). Significance marks: * = *p* < 0.05; *** = *p* < 0.001 (Student *T* test, N = 4).

## Data Availability

The data presented in this study are openly available in the Biostudies repository at DOI 10.6019/S-BSST2833 reference number S-BSST283.
